# General Male Patients’ Experiences When Visiting Public Health Facilities in Limpopo Province, South Africa

**DOI:** 10.1002/puh2.70166

**Published:** 2025-11-14

**Authors:** Lazarros Chavalala, Rachel Tsakani Lebese, Lufuno Makhado

**Affiliations:** ^1^ Department of Public Health, Faculty of Health Sciences University of Venda Thohoyandou South Africa

**Keywords:** experiences, healthcare, male, public, services

## Abstract

**Introduction:**

The quality of healthcare services provided by healthcare workers is crucial and plays an important role in determining patient health‐seeking behaviour and their further engagement with health services. This study explored general male patients’ experiences when visiting public health facilities to use health services in Limpopo Province, South Africa.

**Methods:**

A qualitative exploratory design was employed through in‐depth focus group discussions to gather general men's experiences at visited health facilities. Semi‐structured interviews were conducted through four focus group discussions with 28 purposively selected men. Collected data were audio‐recorded and transcribed verbatim. Tech's eight steps were used to analyse data and guide the development of the main themes and sub‐themes. Trustworthiness was guaranteed via credibility, confirmability, dependability and transferability. Ethical approval was granted by the University of Venda Research Ethics Committee with reference number FHS/21/PH/26/1215.

**Results:**

Participants reported poor service, discrimination, poor communication by health workers, aggressive, rude and disrespectful staff, confidentiality breaches and privacy problems and satisfaction with offered health education.

**Conclusion:**

Services provided at visited public healthcare facilities are poor, and healthcare professionals who assisted participants at visited health facilities did not adhere to ethical guidelines when dealing with patients.

## 1 Introduction

In most countries, public primary healthcare facilities provide free basic primary healthcare services. In this study context, public primary healthcare facilities refer to government‐ and municipal‐owned health facilities that provide basic primary healthcare services to citizens at no cost. The majority of South Africans rely on public primary healthcare facilities to meet healthcare needs, and as of 2022, only 15% of South Africans had medical aid to access private health institutions for health services [1]. In many South African rural areas, public primary healthcare facilities are the only available or easily accessible health service and are most often poorly resourced [2].

Since the inception of Freedom in 1994, there have been efforts to improve the quality of care and make public primary healthcare services accessible in South Africa [3]. The quality of provided services remains key in the utilisation of public primary healthcare facilities. Depending on users’ experiences, the quality of the provided service may encourage or discourage users from interacting with health services. Hompashe et al. [4] state that poor experiences may prevent patients from accessing care. Nkosi [5] indicates that health service users experience services both positively and negatively. Positive experiences include satisfaction with provided services when given attention during attendance, and negative experiences include privacy concerns, drug stock‐outs, staff shortages and inadequate operating hours. A study conducted by Njong [6] established that service users were satisfied with the medical staff's attitude and consultation.

In South Africa, the government has introduced the South African National Integrated Men's Health Strategy 2020–2025 to support and enable men and boys to easily access health services by addressing barriers to health services utilisation among this population group. However, men in South Africa are less likely than women to use health services and tend to be sicker by the time medical help is sought [7]. Due to growing concern about men's poor health‐seeking behaviour, various researchers embarked on studies to uncover barriers to health service utilisation among men. Despite uncovering factors contributing to men's poor health‐seeking behaviours, it remains unclear how men utilising health services, particularly public primary healthcare facilities, have experienced provided services. Moreover, studies conducted by Nkosi [5], Padayachee et al. [8] and Peltzer [9] in South Africa aimed to uncover patient experiences in public health facilities; however, they focused on the general population rather than men, thus leaving the experiences of men in public primary healthcare facilities unknown. Moreover, a study conducted by Njong [10] on patient experiences did include men; however, it could not allow men as participants to express their experiences due to its nature as a quantitative study. Due to the lack of publicly available studies detailing how men have experienced public primary healthcare services in South Africa, the authors embarked on this study to explore the experiences of men who visited public primary healthcare facilities. In this study, we report on 28 interviewed men who have visited public primary healthcare facilities to utilise health services.

## 2 Methods

### 2.1 Study Design

A qualitative exploratory design was used to explore participants’ experiences of provided health services from the visited public health services. This design was chosen because it allowed the researcher to collect detailed information from the participants’ point of view and allowed probing to ensure the depth of information.

### 2.2 Setting

The study was conducted in the Mopani, Vhembe and Capricorn districts of Limpopo Province in South Africa. The districts are predominantly rural, with few people living in townships found near towns in these districts. There are 454 public primary healthcare facilities, 26 community health centres and 41 hospitals in the province [11]. Some citizens walk or travel long distances to access public primary health facilities, whereas some take about 15–20 min to reach the nearest public primary healthcare facility [12]. Many of the citizens in this area depend on public health facilities to access health services.

### 2.3 Population and Sampling

The study was conducted among men aged 18 years and older who were purposively selected and had visited public primary healthcare facilities to use health services less than 3 months ago before this study. The authors only interviewed participants who consented to participate in the study.

### 2.4 Data Collection

In‐depth focus group discussions were conducted in May and June 2024 with 28 men using a semi‐structured interview schedule. The semi‐structured interview schedule was developed by the authors and sent to experts for validation before data collection. The authors approached local tribal authorities to request permission to conduct the study and subsequently asked tribal authorities and local churches to help with mobilising men. Churches and chiefs’ kraals were used to conduct interviews. The first focus group consisted of eight members, the second group had seven members, the third group had seven members and the fourth group had six members. The authors made appointments with focus group members and chose times that were convenient for the participants. On average, a focus group discussion lasted for 1 h. The authors spent 2 weeks conducting focus group discussions. Data saturation was reached during the third focus group discussion; however, the authors added one more group to ascertain if no new ideas were coming through.

### 2.5 Data Analysis

The authors listened to audio recordings of the focus group discussion sessions, transcribed verbatim, read transcripts several times independently and developed codes. However, before the codes were developed, a list of all topics gathered from the transcripts was made, and similar topics were grouped. Furthermore, the authors went back to the data and came up with abbreviations of the topics as codes, which were then written next to relevant parts of the data. The authors then developed words that best described the grouped topics, and those that had relationships were further grouped in their categories. Then themes and sub‐themes were developed. General patients’ experiences related to visitation to public health facilities and suggestions for improving patient experiences when visiting the healthcare facilities were developed as main themes. Poor service delivery, gender disparities and stigma, poor professional conduct, human resources constraints, patient education, staff training and staff motivation came out as sub‐themes.

### 2.6 Trustworthiness

Credibility was established through prolonged engagement with participants when setting appointments to build working relationships to enable participants to be comfortable before actual discussions. During focus group discussions, authors further engaged participants until data saturation was reached. The authors also performed independent data analysis to ensure the credibility of the findings. The authors provided detailed explanations on the selection of participants, and data collection and data analysis were conducted to ensure the dependability of this study's findings. Confirmability was ensured through rigorous and unbiased analysis of data and recording of steps and decisions taken during the study. The authors also attached verbatim quotes to support the unbiased interpretation of data. The authors provided full descriptions of the study's context, participants, sampling methods and data collection sites to ensure the transferability of the study findings to other settings.

### 2.7 Ethical Considerations

The authors requested permission to conduct the study from the chiefs and headmen of the selected communities. This study's ethical approval was granted by the University of Venda Ethics Research Committee (Ethics Approval Number: FHS/21/PH/26/1215). Informed consent was also obtained from individual participants before participating, and they were informed about the study, participating procedures and the right to stop participating at any stage of participation in the study without any consequences.

## 3 Results

### 3.1 Characteristics of Participants

Most of the study's participants were aged 30–39 years, followed by those aged 40–49 years, as shown in Table 1.

**TABLE 1:** Participants’ characteristics.

Group no.	Age	Gender
**Group 1**		
Participant 1	34	Male
Participant 2	26	Male
Participant 3	33	Male
Participant 4	42	Male
Participant 5	32	Male
Participant 6	34	Male
Participant 7	38	Male
Participant 8	43	Male
**Group 2**		
Participant 9	30	Male
Participant 10	39	Male
Participant 11	28	Male
Participant 12	41	Male
Participant 13	30	Male
Participant 14	29	Male
Participant 15	45	Male
**Group 3**		
Participant 16	44	Male
Participant 17	34	Male
Participant 18	25	Male
Participant 19	34	Male
Participant 20	51	Male
Participant 21	24	Male
Participant 22	48	Male
**Group 4**		
Participant 23	38	Male
Participant 24	32	Male
Participant 25	40	Male
Participant 26	46	Male
Participant 27	35	Male
Participant 28	47	Male

Analysed data has led to the formulation of two main themes and three sub‐themes on male patients’ experiences when visiting public health facilities.

### 3.2 General Male Patients’ Experiences When Visiting Public Health Facilities

#### 3.2.1 Poor Service Delivery

The current study findings revealed that participants did not like the quality of health service provided because of the rate at which they attended to patients, and they viewed it as slow. Some patients had to go back home without being helped because the facilities were often full of long queues to see clinicians. The participants also indicated that they had to spend long hours waiting to be seen by a clinician and cited long waiting hours as one of the factors that made them avoid visiting public health facilities (Table 2).

**TABLE 2:** Main theme, sub‐themes and codes on male patients’ experiences at visited public health facilities.

Main theme	Sub‐themes	Codes
**Patients’ experiences related to visitation to public health facilities**	**Poor service delivery**	Patients went back without help Long queues to see clinicians Long waiting period Slow service
	**Gender disparities and stigma**	Treated differently from women Turned away and not allowed to enter the facility
	**Poor professional conduct**	Confidentiality breach Talk badly with patients/shouting at patients Rudeness and lack of respect Blaming and judging patients for an illness Leaving patients unattended Delay in attending to patients
	**Human resources constraints**	Few male nurses/feeling uncomfortable with female nurses Difficult to open for female nurses Few number of staff on duty for the number of patients at the clinic Not enough space and chairs
	**Patient education**	Polite advice and patient education Allow patients to ask questions Few staff members attend to patients with respect
Participants’ suggestions related to improving patient experiences when visiting the healthcare facilities	**Staff training**	Encourage health workers to talk to patients well Motivate employees Change in staff attitude Employee capacity‐building workshops on ethics
	**Staff motivation**	Offer incentives

Participants said:

Sometimes you go to the clinic, but you come back without being helped because the clinic is full of people and there is no space or seats to cover all the people (Participant 1, FDG 1, male, 34‐year‐old);

You can wait for 5 hours or 3 hours if it is less, and you find that nurses sit down and say they are on lunch (Participant 13, FDG 2, male, 30‐year‐old);

If you went there at around 8 in the morning, you would sit until 4 or 2 afternoon, still sitting in the queue without being helped (Participant 6, FDG 1, male, 34‐year‐old);

We sat in the queue for a long time, normally healthcare centres open at 7 or 8 somewhere there. They took about 4 hours until one of the ladies tried to speak, and there was an exchange of words between her and clinicians (Participant 3, FDG 1, male, 33‐year‐old).

#### 3.2.2 Gender Disparities and Stigmatisation

The current study revealed that participants did not like the way they were treated at health facilities during their last visits. They expressed that women were treated better compared to them during emergencies, as women would be attended to quickly, whereas they were made to sit in queues even when they were in severe pain. It was also reported that security personnel would turn them back when visiting the facility without an identity document, while allowing women without an identification document to enter the facilities.

Participants said:

What I have seen is that they quickly run to help women, let's say a woman is pregnant, they are able to quickly help her. But if they look at you and see a man even when you are very sick, you will join the queue (Participant 17, FDG 3, male, 34‐year‐old);

Do you see the security personnel? I am talking from experience, I had to force myself to go inside, and I told them I do not have an Identity document, but if it is a woman, they do not check identity documents, they let them in (Participant 25, FDG 4, 40‐year‐old).

#### 3.2.3 Poor Ethical Conduct

The current study findings revealed that participants reported that healthcare workers did not maintain the confidentiality of patients’ sensitive information and divulged information about what had happened during treatment sessions. The findings also revealed that participants did not like the way healthcare workers spoke to them and described the way healthcare workers spoke to them as rude and lacking respect. Participants reported that clinicians would judge and blame them for having contracted an illness. Moreover, the findings also indicated that clinicians left participants unattended while waiting for help at public health facilities without communicating the reasons for leaving participants as patients unattended.

Participants expressed their experience as follows:

Female nurses do not treat patients’ information with confidentiality in terms of the patient's condition, because after consulting, they talk about your condition with other people, and you thought it was only them that knew your condition (Participant 22, FDG 4, male, 48‐year‐old);

These nurses make jokes about you and the conditions that brought you to the facility, to other patients. When you come out of the consultation room, you will find her busy with what has happened in the consulting room. You will hear her saying, You see, it is necessary to come to check up, you will die. She tells people my problem (Participant 25, FDG 4, male, 40‐year‐old);

When they check and find that you have STI, they will cruelly talk to you, they will say we were not there when you got the disease (Participant 6, FDG 1, male, 34‐year‐old);

When I explained to the lady who was a nurse that I have pains like that, she shouted at me and said that a young child like you, you engage in sexual intercourse, you get this thing by engaging in sex, and you are not using protection, because once you contract this disease it means you were not using protection (Participant 3, FDG 1, male, 33‐year‐old);

It is something that I did not like, waiting for 3, 4 hours without being told the reason we were not attended to, we were not notified to say the reason we are not attending to you, it is because we have this problem, forgive us for that, we were not told anything (Participant 3, FDG 1, male, 33‐year‐old).

#### 3.2.4 Human Resource Constraints

The current study findings revealed that participants indicated that there were more female clinicians than male nurses in the facilities they visited. As males did not feel comfortable being helped by female nurses, it discouraged them from consulting at public health facilities. Participants expressed that it was difficult for them to open up to female nurses during consultation sessions. Participants also reported that there were not enough chairs to accommodate all who came for consultations, and some had to stand while waiting to be assisted.

Participants said:

When I look at the men who work at the clinic are few. If you go to the clinic, you will find that 20 are men and 80 are women, and you will find that I will be helped by women, and it won't be good, that is what we are afraid of as men (Participant 5. FDG 1, male, 32‐year‐old).

I visited a facility, and when I got there, I found that there were many female nurses, but male nurses were not there (Participant 13, FDG 2, male, 30‐year‐old).

#### 3.2.5 Patient Education

This study's findings indicate that despite the participants experiencing challenges described above when visiting health facilities, they were happy with the advice and information offered by nurses during consultation sessions. They reported that the nurses politely gave advice and education.

Participant said:

When I went inside the hospital, I met a female nurse, and according to my views, when I remember what happened on that day, she treated me well, talked to me nicely, and advised me on what I should do going forward to prevent the disease (Participant 7, FDG 1, male, 38‐year‐old).

### 3.3 Participants’ Suggestions for Improved Patient Experience at Visited Public Healthcare Facilities

#### 3.3.1 Staff Training

The findings

The findings revealed that participants suggested healthcare workers should be trained and encouraged to engage patients appropriately. Furthermore, participants suggested that training workshops should also cater for healthcare workers’ ethical conduct and change their negative attitudes towards patients. The training should also happen continuously:

Our nurses, especially public clinics and hospitals, they should be taught to treat patients well, because once you harshly talk with a patient, when the patient is out, he will talk about it that a nurse said these words to me, even the others when they get sick they will be afraid to o clinic because they think the nurses will be hash on them (Participant 2, FDG 1, male, 26‐year‐old).

Participant suggested the following:

Nurses should be trained to treat us well, equally; they should be patient with us, we go to clinics because we are sick (Participant 19, FDG 3, male, 34‐year‐old).

#### 3.3.2 Staff Motivation

The current study findings revealed that participants suggested motivating employees through incentives could improve staff morale and improve the quality of service provided at public primary healthcare facilities.

Participant said:

I think if there can be incentives, and awards, like Nurse of the Year and Nurse of the Month, it could increase their motivation and encourage nurses to treat people well and gain the courage to go to work (Participant 13, FDG 2, male, 30‐year‐old).

## 4 Discussion

On the basis of the study findings, participants experienced challenges during their visits to public primary healthcare facilities and voiced their thoughts on what they thought should be done to give a better experience of health services. Participants reported receiving poor service at visited public primary health facilities due to long queues, lack of space, seeing people going back without being attended to and being treated differently from women. This study's findings concur with the findings of a study conducted in KwaZulu‐Natal by Padayachee et al. [8], who discovered that respondents in their study disagreed that a service provided translated into good quality care. Moreover, this study's findings are also supported by findings from a study conducted by Wei et al. [13], in China, who found that participants in their study were unsatisfied with the quality of treatment received from health workers. This means that participants may hesitate to visit facilities again to use services because of what they have experienced. Participants also reported waiting longer at facilities before they could get assistance because of slow service. This means that patients waited longer than expected at the facility. According to the National Department of Health [14], the South African National Policy on Management of Patient Waiting Time in Outpatient Departments prescribes that the average waiting time is 2 h at South African public primary healthcare facilities. Sastry et al. [15] indicate that persistently long waiting times have been linked to poor medication compliance, skipped appointments, delayed implementation of clinical programmes and low healthcare worker morale.

Participants reported observing health workers being unable to maintain confidentiality and a lack of privacy due to poor infrastructure. The findings are in line with the findings of a study conducted in Denmark by Jensen and Eg [16], who found that participants in their study reported hearing healthcare professionals talk over care, containing information about named patients, their test results, private disclosures and various comments or opinions. These findings imply that healthcare workers do not consider ethical guidelines that prohibit the disclosure of patient information without patient consent when dealing with patients. The Health Professions Council of South Africa guidelines for good practice in the healthcare professions state that healthcare workers must keep patients’ information confidential and seek patient consent to disclose the information and justify where patient information is disclosed. However, poor infrastructure also compromises privacy and affects the confidentiality of patient information. Eg and Jensen [17] state that, because it can be challenging to maintain patient confidentiality during rounds in multiple patient rooms, the physical hospital setting has also been recognised as a potential threat to patient confidentiality.

Facility staff talked badly to patients, acted rudely and disrespected patients. Participants also reported being judged and blamed for contracting illnesses. These findings align with a study conducted by Skär and Söderberg [18] in Sweden, which found that men who were participants in their study reported being disrespected and treated with suspicion by healthcare professionals. This study's findings also concur with the findings conducted by Wessel et al. [19], in Sweden, who found that participants in their study experienced aggressive, rude and arrogant behaviour from health workers. This means that health workers are not taking into consideration their professional code of ethics when dealing with patients and acting based on their values. This also implies that participants may avoid visiting such facilities in the future when they need health services to avoid victimisation by health workers.

Participants were neglected at visited facilities because health workers left them unattended and delayed helping them without any communication, causing delays in offering help. This study's findings are supported by findings from a study conducted by Padayachee et al. [8] in KwaZulu‐Natal, who also found that respondents in their study disagreed that there was effective communication in the visited clinic. Jangland et al. [20] indicate that a lack of communication from health workers makes patients feel abandoned and lonely. This means that health workers cannot communicate effectively with patients on issues affecting service delivery, and as a result, patients feel neglected and are not satisfied with the provided service. According to Sharkiya [21], communication affects the quality of healthcare output, impacts the patient's health and satisfaction and benefits both patients and providers.

However, apart from challenges experienced, participants were pleased with polite health education, showing respect and being given time to ask questions. This study's findings concur with the findings of a study conducted by Brito Fernandes et al. [10] in South Africa, which discovered that respondents in the study were happy with the provided services because their nurses spent enough time with them, gave easy‐to‐understand explanations and provided an opportunity to ask questions or raise concerns during consultation. This implies that despite the challenges participants experienced, there are health workers who adhere to the professional code of ethics and ensure that services are provided with respect.

To improve patient experiences, participants suggested improving the quality of service by encouraging health workers to respect patients, motivating health workers and changing their attitude towards patients. Participants also suggested capacity‐building workshops on ethics to sensitise health workers on the ethics of their conduct when dealing with patients. A study conducted by Laatikainen et al. [22] discovered that healthcare workers who participated in capacity‐building interventions had improved knowledge and skills in the detection and management of non‐communicable disease risk factors.

There is a need to address stereotypes about health workers regarding men. The quality of service provided at health facilities should be re‐examined to identify factors behind poor services in public primary health facilities. Strict adherence to guidelines on ethical conduct for healthcare workers should be strengthened and continuously monitored. Healthcare workers should be equipped with communication skills so that they can effectively communicate with patients. Future research could explore health workers’ perspectives on factors contributing to poor‐quality health services in public primary healthcare facilities. Policymakers could look at policies and ethical guidelines to identify gaps and areas that can be strengthened to improve healthcare service provision.

This study has potential limitations. This study was qualitative and could not establish if there was an association between the type of facility visited and the quality of service participants received at visited facilities. Future studies could employ quantitative methods to measure the association between the type of facility visited and the quality of service received. The authors ran out of funds; therefore, this affected their ability to travel to remote areas and meet certain participants.

## 5 Conclusion

The findings have pointed out that the quality of services provided at visited public healthcare facilities is poor, and healthcare professionals who assisted participants at visited health facilities did not adhere to ethical guidelines when dealing with patients. The participants were viewed differently from female patients, and this influenced the way health workers treated them within the facilities. Despite the challenges that participants went through, there were health workers who were able to provide effective health education with respect and showed commitment to helping participants.

## Author Contributions

**Lazarros Chavalala**: conceptualisation, methodology, investigation, writing – original draft, writing – review and editing. **Rachel Tsakani Lebese**: conceptualisation, methodology, supervision, formal analysis. **Lufuno Makhado**: conceptualisation, supervision, methodology, writing – review and editing.

## Funding

The study was funded by the National Research Foundation.

## Ethics Statement

The authors requested permission to conduct the study from the chiefs and headmen of the selected communities. This study's ethical approval was granted by the University of Venda Ethics Research Committee (Ethics Approval Number: FHS/21/PH/26/1215). Informed consent was also obtained from individual participants before participating, and they were informed about the study, participating procedures, and the right to stop participating at any stage of participation in the study without any consequences.

## Conflicts of Interest

The authors declare no conflicts of interest.

## Data Availability

Data are available on request from the corresponding author.
